# Examining the moderating effect of perceived risk from particulate matter on outdoor sports participants: a theory of planned behavior perspective

**DOI:** 10.3389/fpubh.2024.1340502

**Published:** 2024-01-26

**Authors:** Do Hun Kim, Yunduk Jeong

**Affiliations:** ^1^Department of Sports and Welfare, Korea National University of Transportation, Chungju, Republic of Korea; ^2^College of General Education, Kookmin University, Seoul, Republic of Korea

**Keywords:** perceived risk, particulate matter, theory of planned behavior, outdoor sports, golf

## Abstract

**Purpose:**

This study, drawing from the theoretical framework of the Theory of Planned Behavior (TPB), examines the structural relationship among attitudes, subjective norms, perceived behavioral control (PBC), and behavioral intention. The study focuses on investigating how the perceived risk associated with particulate matter moderates the relationships between “attitudes and behavioral intention,” “subjective norms and behavioral intention,” and “PBC and behavioral intention” within the context of individuals engaged in outdoor sports.

**Design/methodology/approach:**

The data were collected from outdoor sports gatherings facilitated through a popular South Korean sports meetup application. Confirmatory factor analysis was employed to establish the construct validity of the measurement scale, assess factor loadings, averaged variance extracted (AVE), and construct reliability (CR). We also ensured the reliability of the measurement scale through Cronbach’s α analysis. To achieve our research objectives, we utilized structural equation modeling with maximum likelihood estimation to examine the positive relationships under investigation. Additionally, we performed moderation analysis using the statistical software Jamovi.

**Findings:**

The findings demonstrate the significant impacts of attitudes, subjective norms, and PBC on behavioral intention and reveal that perceived risk acts as a moderator, influencing the relationship between PBC and behavioral intention.

## Introduction

As people’s living standards continue to advance and their yearning for spiritual enrichment deepens, there is a burgeoning demand for engagement in outdoor sports activities. Outdoor sports represent a category of sports that occur in natural settings rather than specialized facilities and are characterized by an inherent spirit of adventure and experiential exploration (1). The core essence of outdoor sports lies in venturing beyond urban landscapes, connecting with nature, and participating in activities that involve an element of risk, challenge, and relevance. According to Rocher et al. (2), engagement in outdoor sports has demonstrated substantial benefits for human health. Outdoor sports have proven effective in combating sedentary habits, leading to improved physical well-being, the promotion of healthier lifestyles, better stress management, and enhanced feelings of well-being (3).

In 2022, the worldwide market for Outdoor Sports Apparel reached a valuation of USD 15.87 billion and is projected to grow at a compound annual growth rate (CAGR) of 4.60% from 2023 to 2032 (4). As per Statista, the United States holds the title of the world’s largest outdoor sports market with a valuation of $36.3 billion. Following closely is China’s market at $20.5 billion, trailed by the United Kingdom at $3.3 billion. South Korea secures the fourth position in the market with a valuation of $2.1 billion in 2019. The global outdoor sports market has experienced consistent growth, propelled by a rising focus on healthy lifestyles, the surge in adventure tourism, and an increasing awareness of outdoor recreational activities. The growing community of outdoor sports enthusiasts can significantly contribute to expanding the outdoor sports market. Hence, investigating the determinants impacting individuals’ intention to continue participating in outdoor sports is of paramount importance and substantial value.

This study employs the Theory of Planned Behavior (TPB) to examine the factors that positively influence the inclination to sustain participation in outdoor sports. TPB, an extension of the Theory of Reasoned Action (TRA), is one of the most widely recognized social-psychological frameworks for understanding human behavior (5). Since its introduction in 1991, the TPB has gained broad acceptance among researchers across diverse behavioral disciplines, including but not limited to healthy eating behavior, education, environmentally conscious consumerism, tourism, e-commerce, and sports marketing (6). Researchers in these domains have employed the theory to investigate the factors influencing both intentions and behavior (7). The TPB comprises three primary components: an individual’s attitude toward the behavior, subjective norms pertaining to the behavior, and perceived behavioral control (PBC). Collectively, these factors wield significant influence over an individual’s inclination to engage in a particular behavior (7). Consequently, this study aims to determine if attitudes, subjective norms, and PBC impact the intention to continue participating in outdoor sports.

To address gaps identified in prior TPB research and gain a deeper understanding of the psychology and behavior of amateur athletes, the present study examines how perceived risk related to particulate matter (or air pollution or brown smog) moderates the relationship among the variables under study. Recent reports in Korea have highlighted instances where particulate matter levels exceeded the daily air quality standard. A recent study reveals that approximately 70% of the particulate matter present in the skies over South Korea originates from neighboring Northeast Asian countries, including China. Particulate matter consists of tiny particles invisible to the naked eye (8) and is known to severely affect human health. According to a study by Kwak (9), particulate matter triggers inflammation in the bronchial passages, leading to respiratory issues like chronic bronchitis, allergic rhinitis, and respiratory obstruction. Moreover, it can induce or worsen cardiovascular conditions such as stroke, heart attacks, and irregular heart rhythms. Consequently, individuals might be reluctant to engage in outdoor sports during periods of elevated particulate matter levels. Numerous studies have underscored the myriad advantages associated with outdoor sports, regardless of the presence of particulate matter (10). This study contributes to the existing literature by introducing the moderating variable of perceived risk of particulate matter into an established research model.

In line with the TPB, this study investigates the structural relationships between attitudes, subjective norms, PBC, and behavioral intention. Specifically, the emphasis is on the moderating effects of perceived risk associated with the particulate matter on the relationships between “attitudes and behavioral intention”, “subjective norms and behavioral intention”, and “PBC and behavioral intention”. In contrast to prior TPB research in the sports domain, which primarily investigated the direct impacts of attitudes, subjective norms, PBC on behavioral intention, this study explores the moderating influence of perceived risk between the above relationships. Notably, the moderating impact of perceived risk concerning particulate matter has not been documented in existing literature.

## Literature review and research hypotheses

### Theory of planned behavior

Ajzen’s (11) Theory of Planned Behavior (TPB), an extension of the Theory of Reasoned Action (TRA), initially developed by Ajzen and Fishbein (12), has garnered recognition in research for its efficacy in predicting behavioral intentions. This theory provides a framework for understanding human actions and the immediate motivations that drive them (13). According to Ajzen (11), an individual’s actions are fundamentally rooted in their behavioral intentions. In the TRA framework, intention is perceived as an individual’s mental impetus to exert effort in engaging in a specific behavior (14). In line with the TRA, the behavioral intention is shaped by an individual’s attitudes toward the behavior and the influence of subjective norms.

Attitudes toward behavior pertain to an individual’s overall assessment of engaging in a specific action. These attitudes are shaped by their fundamental beliefs about the potential outcomes of that behavior, known as behavioral beliefs. Behavioral belief represents an individual’s personal perception of the likelihood that engaging in a particular behavior will lead to particular outcomes or result in a particular experience (7). For instance, wearing a mask (the behavior) can protect the body from particulate matter and viruses (the outcome), but it may also be perceived as causing difficulty breathing (the experience). Collectively, these behavioral beliefs are believed to generate either a positive or negative attitude toward the behavior (7). An individual’s attitude toward a behavior tends to be positive when they anticipate favorable outcomes from engaging in that behavior (15).

Subjective norms pertain to the degree to which individuals perceive social pressure when engaging in specific behaviors (11). These norms can be categorized into two categories: exemplary norms, which stem from interpersonal relationships such as family, friends, relatives, and colleagues, and injunctive norms, which are instituted by governmental authorities and employ social incentives to encourage specific actions or informal penalties to discourage others (16). Seonwoo and Jeong (17) affirm that an individual’s intention to continue participating in sports is significantly shaped by the influence of their family, friends, relatives, supervisors, and colleagues. Hence, in the context of this study, subjective norms are closely associated with exemplary norms. Furthermore, subjective norms can be influenced by normative beliefs, which reflect the likelihood of influential figures expressing approval or disapproval of a particular behavior (18).

Nonetheless, several researchers have highlighted limitations in the TRA, indicating its inadequacy in comprehensively explaining certain aspects of individuals’ voluntary behaviors. For instance, individuals engaged in outdoor sports may encounter difficulties upholding their commitment when faced with time constraints stemming from frequent overtime or business trips. In response to these limitations and in accordance with the recommendations of researchers for expanding the TRA, Ajzen (11) introduced the TPB, which incorporates a crucial additional element called Perceived Behavioral Control (PBC). PBC refers to an individual’s perception of the ease or difficulty associated with performing a specific behavior (11). In essence, it assesses an individual’s perception of their ability to manage factors that might constrain their actions in a given situation (19). For instance, when individuals have ample time, resources, and opportunities to navigate a challenging situation, their PBC is expected to be at its highest. Furthermore, it is hypothesized that PBC is influenced by control beliefs, encompassing an individual’s perception of the prerequisites in terms of opportunities and resources necessary to execute a specific behavior (20).

The application of the TPB in a study centered on perceived risks associated with particulate matter is justified for several reasons: (a) Understanding Behavioral Intentions: TPB proves highly suitable for probing into behavioral intentions concerning perceived risks. It encompasses attitudes, subjective norms, and PBC, providing a comprehensive framework to scrutinize individuals’ tendencies and motivations in the context of particulate matter risks. (b) Incorporation of Subjective Norms: TPB integrates subjective norms, which hold significance in the realm of perceived risks. Analyzing how individuals perceive social pressures and norms linked to addressing particulate matter risks yields valuable insights into their behavioral intentions. (c) Consideration of PBC: TPB’s inclusion of PBC facilitates an examination of the perceived ease or difficulty associated with taking specific actions to mitigate particulate matter risks. This aspect is pivotal for comprehending individuals’ perceptions of their capacity to control and manage the associated risks. (d) Predictive Capability: TPB has exhibited its ability to predict outcomes in diverse behavioral scenarios. When employed in the investigation of perceived risks such as particulate matter, researchers can acquire valuable insights into the probability of individuals undertaking specific actions or embracing preventive behaviors in response to these risks. To conclude, the TPB furnishes a sturdy theoretical framework for scrutinizing the intricate interactions among attitudes, subjective norms, PBC, and perceived risks. This renders it especially pertinent and potent in examining behavioral intentions associated with perceived risks from particulate matter.

In recent sports literature, research utilizing the TPB includes the following studies. Bae et al. (21) examined the involvement of adolescents in new sports using the extended TPB, incorporating factors like prior knowledge. The findings revealed a positive impact of the three TPB components—attitude, subjective norm, and perceived behavioral control—on the participants’ intention to engage, subsequently influencing their actual participation behavior. Kang et al. (22) investigated the delinquent behaviors of young athletes by employing the extended TPB along with impulsivity. The analysis through Structural Equation Modeling (SEM) demonstrated that the extended TPB model is sufficient for elucidating deviant behaviors in the realm of sports. The prevalence of TPB in sports research can be attributed to its effectiveness in explaining behavioral intentions, its comprehensive consideration of influencing factors, its predictive power, and its adaptability to diverse sports-related contexts.

### Perceived risk

Perceived risk is a crucial psychological factor in behavioral studies, significantly influencing an individual’s attitude and response. It is defined as the extent of potential loss perceived by an individual (23). In essence, this concept encapsulates an individual’s emotions and understanding of various external threats, highlighting the impact of personal experiences on intuitions and subjective perceptions (24). Perceived risk comprises multiple dimensions and can be evaluated through various indicators (25). According to Stone and Grønhaug (26), perceived risk encompasses physical, psychological, financial, social, functional, and temporal dimensions. Psychological risk pertains to the likelihood of experiencing psychological harm when exposed to a hazard (27). For example, outdoor sports participants feel fear of heights during rock climbing or anxiety related to encountering wildlife during a hiking expedition. Financial risk involves apprehensions about the potential loss of a specific monetary amount (28). For instance, outdoor sports participants spend money on specialized gear, travel expenses, or activity fees with the risk of not gaining the expected satisfaction or outcomes. Social risk is defined as “the likelihood of any unforeseen and unpredictable occurrence, beyond the individual’s control, that results in a substantial decline in their quality of life or standard of living.” For example, outdoor sports participants concern about how friends or family may perceive the individual’s outdoor sports activities or potential social judgments related to skill level or performance. Functional risk relates to concerns and doubts about external hazards interfering with an individual’s ability to execute a plan or engage in an activity. For instance, outdoor sports participants choose the wrong type of equipment for a specific outdoor activity, leading to discomfort or hindrance in performance. Lastly, time risk refers to the squandering or forfeiture of time due to poor choices or unforeseen circumstances (27). For example, outdoor sports participants spend a significant amount of time preparing for and participating in an outdoor sports event, only to find that it did not meet expectations.

Within environmental studies, physical risks are frequently characterized by the tangible ramifications of environmental degradation and fluctuations in climate patterns. Environmental degradation poses a myriad of challenges, with contemporary concerns centered predominantly on the risks associated with particulate matter (27). Particulate matter can be categorized based on particle size, typically falling into the PM10 and PM2.5 categories. Notably, PM2.5 has been found to have a positive correlation with an array of deleterious health outcomes, affecting the respiratory system (e.g., asthma, chronic obstructive pulmonary disease [COPD]), the cardiovascular system (e.g., ischemic heart disease, heart failure), the nervous system (e.g., dementia), cancer (e.g., lung cancer), and mortality, as reported by the EPA (29). Consequently, individuals harbor apprehension regarding the potential health consequences of particulate matter exposure, prompting them to alter their activity routines, including refraining from outdoor engagements (30, 31). Therefore, in the context of this study, outdoor sports participants can encounter an array of risks associated with particulate matter, and their unease concerning the unpredictable consequences can be termed their perceived risk.

### Research hypotheses development

There has been a growing emphasis on examining the impact of attitudes on behavioral intentions. For instance, a study conducted by Rajeh (32) aimed to apply the TPB to identify factors that predict adults’ intentions to enhance their oral health behaviors. The study revealed a positive and significant association between favorable attitudes and stronger intentions. Similarly, Pan and Liu (33) studied the mask-wearing behavior of airline passengers during the COVID-19 pandemic, using the TPB model to investigate the correlation between nine predictive factors and passengers’ intentions to wear masks in the aircraft cabin. The findings underscored the significant influence of attitudes on passengers’ intentions to wear masks during flights amid the pandemic. Furthermore, Wang and Tsai (34) investigated the determinants influencing the behavioral intentions of elementary and junior high school teachers regarding school disaster preparedness. They employed the TPB as the theoretical framework and found a statistically significant direct impact of teachers’ attitudes on their behavioral intentions. Thus, the following hypothesis is formulated:

*H1: Attitudes positively influence behavioral intention.*

A substantial body of research has established a robust connection between subjective norms and behavioral intentions. For instance, a study by Jeong et al. (35) investigated the decision-making process of sports enthusiasts during the SARS-CoV-2 pandemic, revealing the favorable impact of subjective norms on the intention to attend sports events. Carfora et al. (36) conducted a psychological analysis focusing on the factors influencing attitudes toward and purchases of organic products using the TPB framework. Their findings indicated that subjective norms played a significant role in predicting the intentions of Italians to purchase organic milk. Furthermore, Yadav and Pathak (37) delved into the factors that shape consumers’ environmentally friendly purchasing behavior, demonstrating the supportive role of subjective norms in influencing consumers’ intentions to opt for green products. Hence, we propose the following hypothesis:

*H2: Subjective norms positively influence behavioral intention.*

Existing research consistently demonstrates a positive correlation between PBC and behavioral intention. For instance, Lavuri (38) investigated the factors that contributed to the propensity of millennials to purchase eco-sustainable products in emerging markets, revealing a statistically significant and positive effect of PBC on their purchasing intentions. A study by Zarei et al. (39) developed a robust model that comprehensively explains the formation of pro-environmental behavioral intentions. Their research revealed that PBC exerted a positive role in shaping behavioral intention. Similarly, Ateş (40) sought to elucidate the determinants of pro-environmental behaviors within the framework of the TPB. The findings elucidated a direct impact of PBC on intentions to engage in pro-environmental actions. Consequently, the proposed hypothesis is:

*H3: Perceived behavioral control positively influences behavioral intention.*

The concept of perceived risk, serving as a moderating factor, becomes evident when it influences the manifestation of behavioral intention. In the realm of tourism marketing, for example, individuals with lower risk perceptions exhibit a greater propensity to engage in repeat visits or recommend the destination to others compared to travelers with higher levels of perceived risk (41). In their seminal work, Campbell and Goodstein (42) established the moderating effect of perceived risk on consumers’ product assessments. Their research suggested that heightened risk perception tends to induce a more cautious consumer behavior, while reduced risk perception tends to make consumers more receptive to positive cues, resulting in more favorable product evaluations (42). Further research by Tavitiyaman and Qu (43) examined the moderating role of perceived risk in shaping the connection between overall satisfaction and behavioral intention. Travelers who exhibited a reduced sense of risk associated with natural disasters tended to form a more favorable perception of the destination, experienced increased overall satisfaction, and exhibited a greater proclivity towards positive behavioral intentions. This was in contrast to those who perceived a higher level of risk. Building on this literature review, we propose the following research hypotheses:

*H4-1: Perceived risk moderates the influence of attitudes on behavioral intention.*

*H4-2: Perceived risk moderates the influence of subjective norms on behavioral intention.*

*H4-3: Perceived risk moderates the influence of perceived behavioral control on behavioral intention.*

**Figure 1:**
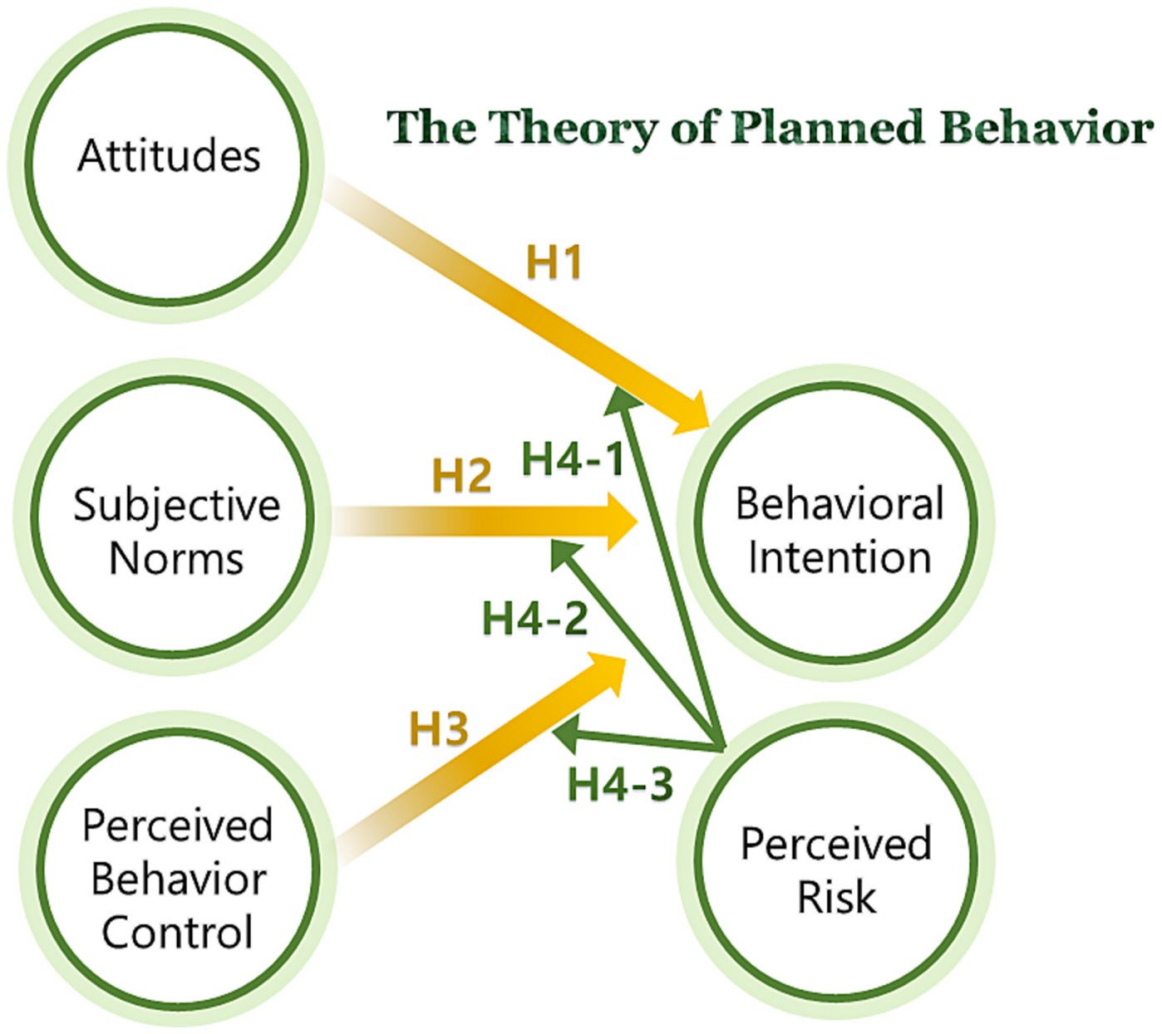
Proposed conceptual model.

Drawing from the existing body of literature, the present study employed the conceptual framework illustrated in Figure 1.

## Method

### Sampling and date collection

We conducted a survey targeting amateur athletes who regularly participated in various outdoor sports activities such as running, golf, cycling, sailing, surfing, marathons, and skydiving. From early March to early April 2023, the researchers reached out to these athletes through a popular South Korean sports meet-up application. First, we explained the study’s purpose, nature, and survey methodology to the participants and obtained their consent. Subsequently, we were invited to join the group chat room of each meet-up, where we once again elaborated on the study details and shared a link for the Google Form survey. Participants expressed their consent by marking a “V” on the questionnaire and were informed of their right to discontinue the survey at any point. Consenting participants completed self-administered questionnaires. We employed a convenience sampling method, distributing a total of 270 questionnaires. We collected 252 valid responses for data analysis. The demographic characteristics included basic personal details such as gender (80.2% male and 19.8% female); age (28.6% aged 50 and above, 27% in their 40s, and 22.2% in their 20s and 30s); and education level (41.3% university graduates, 30.2% high school graduates, 15.1 with an associate degree, and 13.5% graduate degree holders).

### Measure

The questionnaire included questions related to attitudes, subjective norms, PBC, perceived risk, and behavioral intention. All measurement scales used in this study were derived from established scholarly literature and had undergone rigorous validation in prior research investigations. Attitudes were assessed using three items adapted from Ajzen (44) and Kim et al. (15). The scales were “boring-exciting,” “worthless-valuable,” and “unpleasant-pleasant”, with interval scales ranging from 1 to 5. Subjective norms were assessed using three items from Ajzen (44) and Yu and Jeong (6). PBC was assessed using three items adapted from Han et al. (45) and Seonwoo and Jeong (17). The behavioral intention was assessed using three items from Seonwoo and Jeong (17) and Yu and Jeong (6). Perceived risk was assessed using four items adapted from Kim et al. (46) and Stenlund et al. (47). Responses were collected using a 5-point Likert scale ranging from 1 (strongly disagree) to 5 (strongly agree). The content validity of the study instruments was assessed by a panel consisting of two experts in the sociology of sports and one specialist in environmental studies. These panelists evaluated the relevance, representativeness, and clarity of the items for each construct. Incorporating their valuable feedback, the initial questionnaire underwent refinements and enhancements, resulting in the final version used for dissemination. Furthermore, before administering the survey, requisite approval was sought and obtained from the university’s Institutional Review Board.

### Validity and reliability

This research utilized confirmatory factor analysis (CFA) with maximum likelihood estimation in AMOS to substantiate the structure of the measurement model. The goodness-of-fit indicators for the CFA, which included x^2^/*df* = 1.405, NFI = 0.968, RFI = 0.954, TLI = 0.986, and RMSEA = 0.039, consistently fell within the recommended thresholds as suggested by Hooper et al. (48).

This investigation computed factor loadings, construct reliability (CR), and the averaged variance extracted (AVE) to assess convergent validity based on the measurement model. As presented in Table 1, all factor loadings (ranging from 0.815 to 0.967) exhibited statistical significance (*p* < 0.001) and exceeded the established threshold of 0.50, as recommended by Hair et al. (49). Furthermore, all CR values (ranging from 0.810 to 0.920) surpassed the requisite minimum of 0.7, and all AVE values (ranging from 0.588 to 0.792) surpassed the recommended threshold of 0.5, as proposed by Tabachnick and Fidell (50). Given that all CR and AVE values met or exceeded the prescribed criteria, the convergent validity of the measures was duly established.

**Table 1:** Confirmatory factor analysis results.

Scale items	Standardized loadings	CR	AVE	Cronbach’s α
**Attitudes**				
Playing outdoor sports is extremely boring—extremely exciting	0.941	0.920	0.792	0.964
Playing outdoor sports is extremely worthless—extremely valuable	0.967			
Playing outdoor sports is extremely unpleasant—extremely pleasant	0.936			
**Subjective norms**				
The people in my life (e.g., family/friends) would be in favor of me doing outdoor sports.	0.815	0.810	0.588	0.893
The people in my life (e.g., family/friends) would support me doing outdoor sports.	0.826			
The people in my life (e.g., family/friends) would encourage me to do well in outdoor sports.	0.943			
**Perceived behavior controls**				
It is entirely up to me whether I do outdoor sports.	0.820	0.889	0.727	0.914
If I want, I can do outdoor sports.	0.927			
I have enough time and money to do outdoor sports.	0.907			
**Behavioral intentions**		0.887	0.724	0.948
I will try to continue doing outdoor sports.	0.931			
I intend to continue doing outdoor sports.	0.912			
I am willing to devote money and time to do outdoor sports.	0.941			
**Perceived risk**				
I’m worried about particulate matter causing respiratory problems when I play outdoor sports.	0.887	0.874	0.635	0.925
I’m worried that particulate matter will cause cardiovascular disease (heart and major arteries) when I play outdoor sports.	0.904			
I’m worried about particulate matter triggering allergic rhinitis when I play outdoor sports.	0.852			
I’m worried about particulate matter causing lung cancer when I play outdoor sports.	0.839			

x^2^/df = 1,405 NFI = 0.968, RFI = 0.954, TLI = 0.986, RMSEA = 0.039.

To ascertain the adequacy of discriminant validity, it was imperative that the diagonal elements within Table 2 exceed the off-diagonal elements, a condition that was indeed met. Furthermore, comparing all correlation coefficients with the square roots of AVE demonstrated a favorable level of discriminant validity.

**Table 2:** Correlations between constructs.

Construct	Attitudes	Subjective norms	PBC	Behavioral intentions	Perceived risk
Attitudes	**0.890**				
Subjective norms	0.611**	**0.767**			
PBC	0.283**	0.391**	**0.853**		
Behavioral intentions	0.585**	0.631**	0.493**	**0.851**	
Perceived risk	−0.627**	−0.558**	−0.140*	−0.501**	**0.797**

**p < 0.01, *p < 0.05.

Reliability assessments, measured by Cronbach’s alpha, for the constructs of attitudes, subjective norms, PBC, behavioral intentions, and perceived risk (ranging from 0.893 to 0.964), all surpassed the recommended threshold of 0.7, affirming the robustness of the measurement instruments as per the guidelines outlined by Fornell and Larcker (51) (refer to Table 1 for details).

## Results

### Model fit and structural model

**Figure 2:**
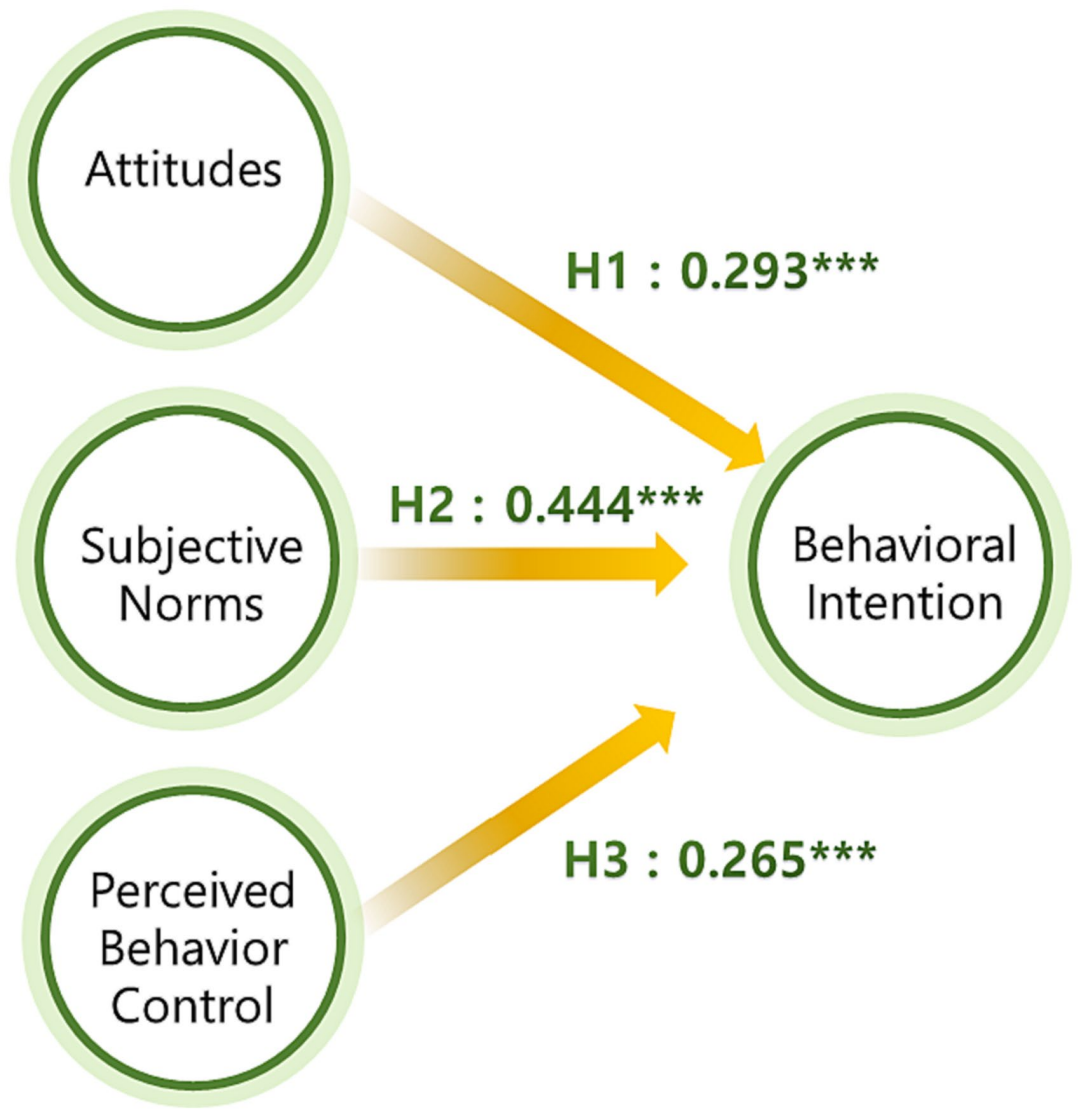
Structural model results. ***p < 0.001.

This study employed Structural Equation Modeling (SEM) to examine the proposed associations. All goodness-of-fit indicators for the model confirmed its acceptability (x^2^/*df* = 1.322, NFI = 0.980, RFI = 0.968, CFI = 0.995, RMSEA = 0.036). This model served as the basis for testing hypotheses 1, 2, and 3. As depicted in Figure 2, the association between attitudes and behavioral intention (H1) was found to be statistically significant (0.293, *p* < 0.001). Notably, significant pathways were observed from subjective norms to behavioral intention (0.444, *p* < 0.001), thereby supporting H2. Additionally, the effect of PBC on behavioral intention was significantly positive (0.265, *p* < 0.001), thus reinforcing hypothesis H3.

### Tests of moderating effect

As hypothesized in H4-1, both attitudes (*Z* = 5.15, *p* < 0.001) and perceived risk (*Z* = −2.57, *p* < 0.05) exhibited significant effects on behavioral intention. However, their interaction did not yield a significant effect on behavioral intention. Consequently, it can be concluded that perceived risk did not moderate the relationship between attitudes and behavioral intention. Thus, H4-1 was not supported.

As hypothesized in H4-2, subjective norms (*Z* = 8.664, *p* < 0.001) had a significant effect on behavioral intention. However, perceived risk and its interaction with subjective norms did not demonstrate significant effects on behavioral intention. Therefore, the perceived risk did not moderate the relationship between subjective norms and behavioral intention. Thus, H4-2 was not supported.

**Figure 3:**
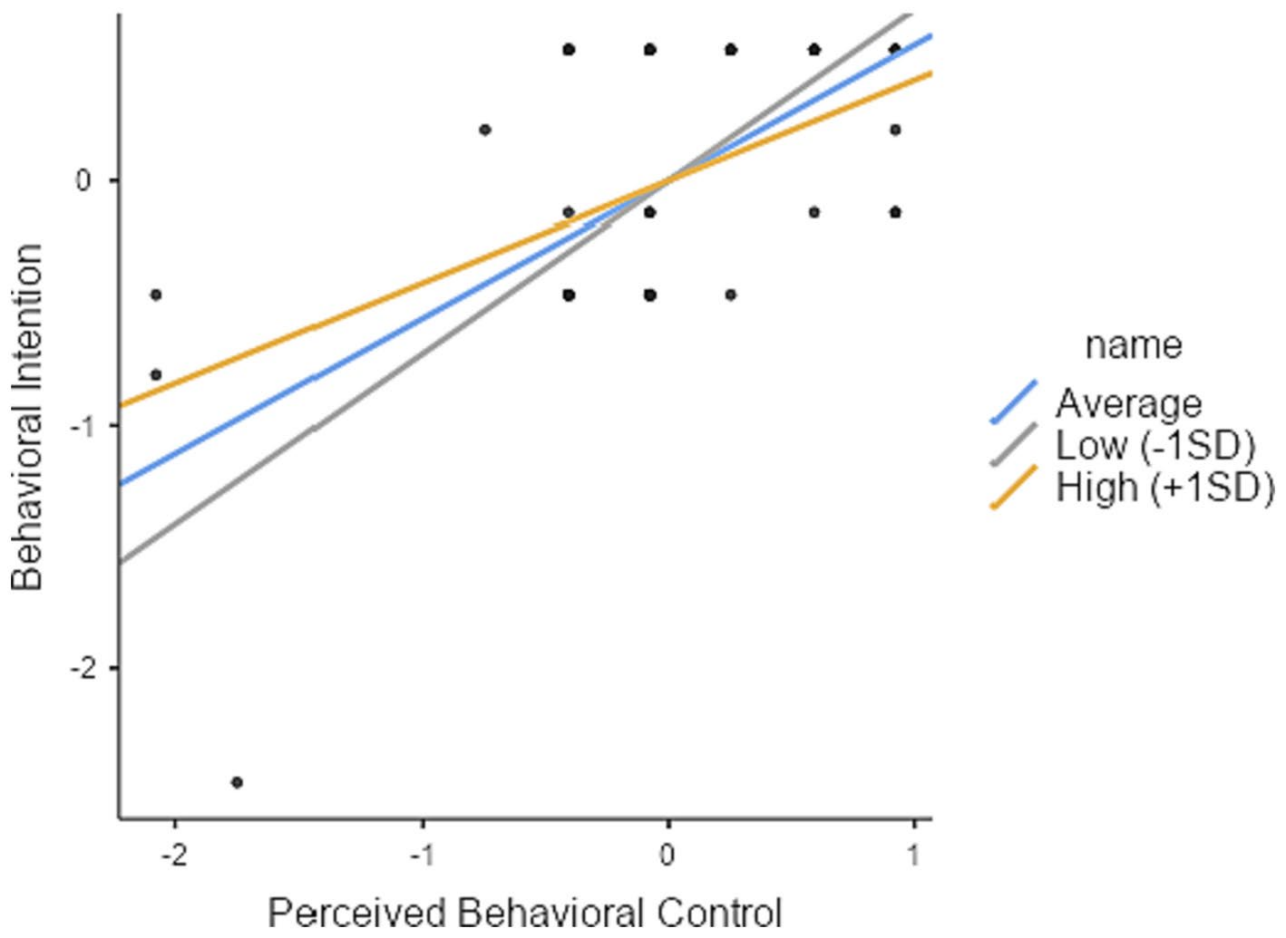
Simple slope plot of the moderating variable for perceived risk (H4-3).

As hypothesized in H4-3, PBC (*Z* = 12.05, *p* < 0.001) exerted a significant influence on behavioral intention. Perceived risk, on the other hand, did not demonstrate a significant impact on behavioral intention. However, it is important to note that their interaction (*Z* = −4.197, *p* < 0.001) had a statistically negative effect on behavioral intention, thereby supporting H4-3 (see Table 3). The independent variable exhibits a positive sign, the moderator variable demonstrates a negative sign, and their interaction also displays a negative sign, indicating the presence of a buffering effect. Subsequently, this study conducted a simple slope analysis to confirm the variations in the slope between the independent and dependent variables based on the moderating variable, categorizing the moderating variable into three groups: low (mean − 1SD), average, and high perceived risk (mean + 1SD). The results are shown in Table 3. The analysis found that across all three groups (low, average, and high perceived risk), the variable of PBC had a significant effect on behavioral intention (*Z* = 12.05, 10.38, 8.85, respectively; *p* < 0.001 in all cases). In Figure 3, it is evident that as one transitions from low to high clustering graphs, the slope gradually attenuates. This observation indicates that the moderating variable serves to diminish the impact of the independent variable on dependent variable.

**Table 3:** Moderating effects of perceived risk.

			Estimate	SE	Z	*p*
H4-1	Moderation estimates	Attitudes	0.2759	0.0535	5.15	< 0.001
		Perceived risk	−0.0777	0.0302	−2.57	0.010
		Attitudes × perceived risk	−0.0492	0.0380	−1.29	0.196
H4-2	Moderation estimates	Subjective norms	0.43412	0.0501	8.664	< 0.001
		Perceived risk	0.00662	0.0284	0.233	0.816
		Subjective norms × perceived risk	−0.05976	0.0368	−1.626	0.104
H4-3	Moderation estimates	PBC	0.5583	0.0455	12.279	< 0.001
		Perceived risk	−0.0236	0.0254	−0.927	0.354
		PBC × Perceived risk	−0.1298	0.0309	−4.197	< 0.001
	Simple slope estimates	Average	0.558	0.0464	12.05	< 0.001
		Low (-1SD)	0.704	0.0678	10.38	< 0.001
		High (+1SD)	0.413	0.0467	8.85	< 0.001

## Discussion

### Theoretical implications

The study’s findings underscore the direct and positive impact of subjective norms on shaping behavioral intentions. Engaging in outdoor sports demands significant time and financial resources and entails a higher risk of injury compared to indoor sports. Consequently, participants place substantial importance on the support and encouragement they receive from their loved ones. This support serves as validation for their decision to participate in outdoor sports, consequently amplifying their behavioral intentions. In the context of our study, it can be argued that harnessing social pressures stemming from the perceptions and expectations of others can effectively strengthen behavioral intentions, a conclusion that aligns with recent research findings. For instance, Ng (52) extended the Theory of Planned Behavior (TPB) to investigate the influence of risk perception on disaster preparedness behavior and identified subjective norms as a predictor of intention. Similarly, a study by Si et al. (16) identified subjective norms as a determinant of individuals’ intention to engage in water conservation.

Furthermore, while Ajzen (11) asserts that attitudes are typically the most reliable predictors of intention, this study reveals that subjective norms have emerged as the most influential factor in forecasting behavioral intention—an outcome consistent with previous research where attitudes frequently occupied the primary predictive position. For instance, Kim and Hwang (53) integrated the norm activation model and TPB to elucidate the formation of eco-friendly behavioral intentions and found attitudes to be the strongest predictor. Our study corroborates this in the realm of sports literature, as subjective norms exhibited the most substantial effect size, consistent with recent research in sports. For example, Yu and Jeong (6) investigated the structural relationships among attitudes, subjective norms, perceived behavioral control (PBC), and career pursuit intentions among aspiring esport athletes. Their findings identified subjective norms as the most influential factor in predicting career pursuit intentions. This outcome underscores the pivotal role played by family and close friends in the decision-making process within the sports context.

The findings of this study revealed a significant association between attitudes and the intention to participate in outdoor sports, suggesting that individuals with more favorable views regarding outdoor sports are more likely to express an intention to participate. When individuals perceive outdoor sports as beneficial or enjoyable, whether based on past or current experiences, it naturally fosters a positive attitude toward participating in these activities. In essence, the relationship between a positive attitude and behavioral intention is likely mediated by positive experiences. This finding is in line with recent research. For instance, Lavuri (38) investigated factors influencing millennials’ purchasing intentions regarding eco-sustainable products and found a direct positive impact of millennial green attitudes on their purchase intentions. Similarly, Das et al. (54) investigated the determinants of social distancing behavior during the COVID-19 outbreak, demonstrating that attitudes toward social distancing influenced the intention to adhere to social distancing measures. While the relationship between attitudes and behavioral intention is statistically significant, it is important to note that, in this study, attitudes do not emerge as the most potent predictor of intentions. Despite the strength of this association being surpassed by the relationship between subjective norms and behavioral intention, both attitude and subjective norms play significant roles in predicting behavioral intentions.

The findings illuminate the significance of PBC as an integral factor linked to behavioral intentions. While PBC exhibited the weakest correlation with behavioral intention compared to attitudes and subjective norms, it still displayed a positive and statistically significant relationship. This correlation underscores the pivotal role of individuals’ perceptions about their abilities to engage in outdoor sports activities. Put simply, when participants in outdoor sports believe that they have sufficient resources to partake in these activities, their inclination to participate is strengthened. Conversely, doubts regarding the availability of opportunities, time, or financial means may undermine behavioral intention. In essence, the perceived level of ease or difficulty in engaging in outdoor sports can substantially influence behavioral intent.

Several researchers have underscored the importance of PBC in predicting behavioral intention. For instance, Raimondo et al. (55) investigated the factors influencing millennials’ intentions and behaviors related to reduced plastic consumption. Structural equation modeling revealed a significant influence of PBC in shaping the intention to reduce the use of plastic drinking bottles. Similarly, Parveen and Ahmad (56) examined how individuals’ intentions to consume environmentally friendly products, contributing to reduced urban air pollution, influenced their actual behavior. Their findings indicated that a higher level of PBC regarding the use of eco-friendly products predicted the intention to consume products with lower pollution levels. Consequently, the significance of PBC in enhancing the intent to participate in outdoor sports should not be underestimated.

The findings of the present study indicate that perceived risk serves as a moderator, influencing the relationship between PBC and behavioral intention. Specifically, this relationship tends to weaken for individuals with higher levels of perceived risk. In essence, the impact of PBC on behavioral intention is less pronounced among those who perceive particulate matter as a significant concern compared to those who perceive it as less severe. Individuals with heightened perceived risk may have had adverse experiences related to particulate matter or other forms of environmental pollution. Particulate matter is associated with health issues such as allergic rhinitis, allergic conjunctivitis, respiratory problems, otitis media, hair loss, diabetes, and high blood pressure. Consequently, those who have experienced these health issues may exhibit greater sensitivity to particulate matter and potentially harbor reservations about participating in outdoor sports. These findings underscore the paramount importance of considering particulate matter in the context of outdoor activities.

Furthermore, while prior research has explored the relationships between perceived risk and attitudes (57, 58), there has been a dearth of comprehensive investigations into the moderating role of perceived risk within the framework of the TPB. Our study systematically explores these moderating effects of perceived risk, shedding light on its role in modulating the relationship between PBC and behavioral intention. This scholarly contribution offers novel insights into the concept of perceived risk. Given the evident significance of perceived risk regarding particulate matter in predicting behavioral intention, it is imperative for future research endeavors to prioritize in-depth exploration of its impact.

### Practical implications

The findings of this investigation bear significant implications as they can serve as a catalyst for raising public awareness regarding the health risks linked to particulate matter, an issue of paramount societal importance. It is pertinent to note that despite the general awareness among the public about the risks posed by particulate matter during outdoor physical activities, both national and local governmental entities have yet to implement sufficiently robust protective measures. This lack of comprehensive protective measures exacerbates the health risks faced by individuals. Consequently, an immediate and pressing need exists for the implementation of enhanced protective measures and the promotion of increased awareness concerning the perils associated with particulate matter, as underscored by Choi and Bum (10).

In the pursuit of mitigating the presence of particulate matter, it is incumbent upon both national and local governments to undertake several essential initiatives: (1) Significantly Reduce Diesel Vehicle Numbers and Promote Public Transportation: A pivotal measure involves a substantial reduction in the population of diesel-powered vehicles, a known source of particulate matter emissions. Concurrently, there should be a concerted effort to invigorate public transportation systems. (2) Drastically Curtail Coal Power Plants: It is imperative to halve the number of coal power plants, given their role as a prominent contributor to particulate matter emissions. (3) Enforce Stringent Regulations on Particulate Matter Emissions from Corporations: Robust management of particulate matter emissions from corporate entities is essential to curbing their prevalence. (4) Improve Energy Efficiency and Embrace Renewable Energy Sources: Measures should be taken to enhance energy efficiency and expand the adoption of renewable energy sources, thereby reducing reliance on sources that contribute to particulate matter. (5) Establish Collaborative Reduction Targets: National and local authorities should collaborate to set reduction targets and commit to a collective effort to mitigate particulate matter levels. By diligently pursuing these comprehensive measures, governments can significantly contribute to reducing particulate matter levels and, in turn, safeguarding public health.

Sports companies and participants have proactively engaged in a spectrum of initiatives aimed at mitigating particulate matter. These are: (1) Promoting eco-friendly Transportation: Encouraging individuals to opt for walking as an eco-conscious mode of transportation when traveling to nearby sports venues. (2) Minimizing Vehicle Idling: Advocating against prolonged idling while driving, thereby reducing emissions that contribute to particulate matter. (3) Optimizing the Indoor Environment: Ensuring that indoor sports facilities are maintained at an appropriate heating temperature, typically 20 degrees Celsius. This measure serves the dual purpose of minimizing energy consumption and emissions. (4) Greening Outdoor Spaces: Initiating programs for planting foliage in outdoor sports facilities to improve air quality and create a more pleasing environment. (5) Sustainable Equipment Choices: Promoting the responsible use of plastic materials in acquiring sports equipment and adopting reusable grocery bags, aligning with sustainability objectives. These collective efforts not only contribute significantly to the reduction of particulate matter but also enhance the overall safety and enjoyment of outdoor sports activities in an environment characterized by fresh, clean air.

## Conclusion

This study, rooted in the theoretical framework of the TPB, explores the structural connections among attitudes, subjective norms, PBC, and behavioral intention. Specifically, our investigation explores the moderating influence of perceived risk related to particulate matter on the associations between “attitudes and behavioral intention,” “subjective norms and behavioral intention,” and “PBC and behavioral intention” within the context of individuals engaged in outdoor sports. Data were collected from outdoor sports gatherings organized through a prominent sports meetup application in South Korea. We employed confirmatory factor analysis to establish the construct validity of the measurement scale, assess factor loadings, averaged variance extracted (AVE), and construct reliability (CR). The measurement scale’s reliability was further confirmed using Cronbach’s α analysis. To fulfill our research objectives, we leveraged structural equation modeling with maximum likelihood estimation to investigate the examined positive relationships. Additionally, moderation analysis was conducted using the statistical software Jamovi. The findings underscore the significant impacts of attitudes, subjective norms, and PBC on behavioral intention, while also revealing that perceived risk exerts a moderating effect, influencing the relationship between PBC and behavioral intention. Therefore, the government will need to exert substantial efforts to mitigate particulate matter levels.

Notwithstanding the valuable contributions of this study, it is essential to acknowledge several limitations that merit consideration in future research endeavors. Firstly, this investigation did not explore the antecedents of attitudes, subjective norms, and PBC. Existing scholarship has revealed the potential influence of various factors on these key determinants of behavioral intention. Therefore, it is incumbent upon future studies to scrutinize these potential antecedents, thereby contributing to a more comprehensive understanding of the underpinnings of attitudes, subjective norms, and PBC. Secondly, a noteworthy opportunity exists to explore additional moderating factors that may further enrich the theoretical framework. Investigating potential moderating factors would equip researchers with deeper insights into the complexities of the relationships among the core constructs, ultimately enhancing the model’s predictive power. Lastly, it is imperative to acknowledge that the current study was conducted within the context of South Korea. Consequently, the findings may not be universally applicable to other countries or cultural settings. Cultural nuances and contextual differences can profoundly influence the validity and generalizability of research outcomes. Therefore, it is both pertinent and valuable to extend the examination of the model to diverse cultural settings, ensuring that its applicability transcends national boundaries and offers relevant and adaptable insights to various sociocultural contexts. This approach contributes significantly to the robustness and external validity of the research.

## Data availability statement

The original contributions presented in the study are included in the article/supplementary material, further inquiries can be directed to the corresponding author.

## Ethics statement

The studies involving humans were approved by Institutional Review Board of Korea National University of Transportation. The studies were conducted in accordance with the local legislation and institutional requirements. The participants provided their written informed consent to participate in this study.

## Author contributions

DK: Conceptualization, Data curation, Formal analysis, Funding acquisition, Investigation, Methodology, Writing – original draft. YJ: Project administration, Resources, Software, Supervision, Validation, Visualization, Writing – review & editing.
